# Disruption of the pro-inflammatory, anti-inflammatory cytokines and tight junction proteins expression, associated with changes of the composition of the gut microbiota in patients with irritable bowel syndrome

**DOI:** 10.1371/journal.pone.0252930

**Published:** 2021-06-11

**Authors:** V. Ivashkin, Y. Poluektov, E. Kogan, O. Shifrin, A. Sheptulin, A. Kovaleva, A. Kurbatova, G. Krasnov, E. Poluektova

**Affiliations:** 1 I.M. Sechenov First Moscow State Medical University (Sechenov University), Moscow, Russian Federation; 2 Engelhardt Institute of Molecular Biology, Russian Academy of Sciences, Moscow, Russia; University of Nebraska Medical Center, UNITED STATES

## Abstract

**Background:**

Irritable bowel syndrome (IBS) is a pathologic condition characterized by changes in gut microbiome composition, low-grade inflammation, and disruption of intestinal wall permeability. The interaction between the gut microbiome and the disease manifestation remains unclear. The changing of tight junction proteins and cytokines expression throughout the gastrointestinal tract in IBS patients has not been studied yet.

**Aim of the study:**

To assess the changes of gut microbiome composition, tight junction proteins, and cytokines expression of intestinal mucosa from the duodenum to the distal part of the colon in IBS patients and healthy volunteers.

**Methods:**

In 31 IBS patients (16 patients with IBS-D; 15 patients with IBS-C) and 10 healthy volunteers the expression of CLD-2, CLD-3, CLD-5, IL-2, IL-10, and TNF-α in mucosal biopsy specimens was determined by morphological and immune-histochemical methods. The qualitative and quantitative composition of the intestinal microbiota was assessed based on 16S rRNA gene sequencing in both groups of patients.

**Results:**

The expression of IL-2 and TNF-α was significantly increased in IBS patients compared with the controls (p<0.001), with a gradual increase from the duodenum to the sigmoid colon. The expression of IL-10, CLD-3, and CLD-5 in mucosal biopsy specimens of these patients was lower than in the control group (p<0.001). Increased ratios of *Bacteroidetes* and decreased ratios of *Firmicutes* were noted in IBS patients compared to healthy volunteers (p<0.05).

**Conclusion:**

IBS patients have impaired gut permeability and persisting low-grade inflammation throughout the gastrointestinal tract. Changes in the gut microbiota may support or exacerbate these changes.

## Introduction

Irritable bowel syndrome (IBS) is a common gastrointestinal disorder that is characterized by abdominal pain, bloating, change in stool frequency or form. IBS negatively affects patient’s quality of life and places a considerable financial burden on healthcare systems [1]. IBS traditionally has been conceptualized as a functional brain-gut axis disorder [2]. Nevertheless, disruption of intestinal permeability, changes in cytokine profile, and inflammation in the mucous membrane of the intestinal wall leads to symptom manifestation in IBS patients [3–5].

The intestinal barrier has three levels: the pre-epithelial (mucus on the epithelium surface), epithelial (tight junctions (TJs), high capability of epithelial regeneration), and post-epithelial (immune cells) [3]. The intestinal microbiota can also be considered as a part of the intestinal barrier in the pre-epithelial level [6]. The mucus layer limits the contact of the intestinal contents with epithelium [7]. TJs are formed from transmembrane proteins, such as occludin, claudins (CLDs), junctional adhesion molecules (JAMs), and tricellulin. Main function of TJs is to regulate the passage of water and electrolytes through the intercellular spaces, prevent the translocation of antigens, microorganisms, their toxins, and their contact with immune cells [8–10]. The intestinal microbiota metabolites, such as short-chain fatty acid (SCFA), modulate mucus layer strength, and immune response (post-epithelial level) [11].

The increase of intestinal permeability in IBS patients is determined by changes in synthesis of transmembrane proteins—occludins and CLDs [12–14]. A decrease of CLD-1 expression has been found in patients with IBS-D, whereas an increase of this protein expression has been noted in IBS-C patients [13]. Reduced expression of occludin, E-cadherin, and zonula occludins in the cecum has been observed in both groups of IBS patients [14, 15].

Various results have been reported for cytokines at the mucosal level in IBS patients: increased expression of IL-1b [16], TNF-a [17], decreased levels of IL-10 [4, 18] and IL-8 [19]. These changes can lead to the activation of the immune response, cytokines disbalances, and low-grade mucosal inflammation.

Changes in microbiota composition (low microbial diversity, reduction of SCFA producing bacteria) were reported in patients with IBS [20]. Also, it has been studied that conditions altering normal microbiome composition (small intestinal bacterial overgrowth, dysbiosis, and infectious disease) may be predisposing or “trigger” factors for the IBS onset.

The decrease of TJ protein expression and the number of short-chain fatty acid-producing bacteria can lead to the disruption of the intestinal barrier permeability, formation of inflammatory changes in the intestinal wall, changes in sensitivity, and motility, and IBS symptoms.

This study is aimed to assess changes of pro-inflammatory, anti-inflammatory cytokine profiles, tight junction’s proteins expression in intestinal mucosa from the duodenum to the distal part of the colon, and intestinal microbiota composition in IBS patients and healthy volunteers.

## Materials and methods

### Patients cohort

Participants were recruited during their medical treatment at the Sechenov University, Moscow, Russia since June 8 to Septembler 11 2016. Initially, we included in the study 77 patients with symptoms of IBS according to the Rome IV criteria. Non-functional causes for the symptoms were excluded after a detailed evaluation of medical history, physical examination, extensive panel of blood tests, stool analysis, and colonoscopy with biopsies. The exclusion criteria were patients younger than 18 years, older than 59 years, patients with organic bowel disease, organic disease of pancreas and gallbladder, renal disease, hepatic insufficiency, mental illness significantly impairing self-report (schizophrenia, bipolar disorder, or epilepsy), and receiving antibiotic or probiotic therapy for the previous 6 months. After analyzing the medical history and conducting laboratory and instrumental studies, 46 patients were excluded. The final size of the cohort was 31 patients. Additionally, ten healthy volunteers were included in the control group. Written informed consent was obtained from all participants.

The study protocol was approved by the ethics committee of Sechenov University, Moscow, Russian Federation.

#### Sample size calculation

We did not perform a sample size calculation before the study initiation due to its complexity. Based on an analysis of the previous studies, we found a large difference in the levels of pro-inflammatory cytokines in IBS patients compared with controls. Using G*Power software [21, 22] we calculated the analysis power, which reached 0.99 in all cases and, therefore, the sample size is sufficient for achieving statistical significance.

### Immunological tests

The expression of pro-inflammatory (interleukin-2 (IL-2), tumor necrosis factor-α (TNF-α)) and anti-inflammatory cytokines (interleukin-10 (IL-10)), as well as tight junction proteins (CLD-2, CLD-3, and CLD-5) in the mucosal biopsy (duodenum, ileum, cecum, and sigmoid colon) were examined.

Esophagogastroduodenoscopy was performed with EG-250 WR5 gastroscope (Fujinon, Japan). Colonoscopy was performed with EVIS Exera II CLV-180 colonoscope and OEV-191H video monitor (Olympus, Japan). During endoscopic examinations, 11 fragments of the mucosal specimens were obtained: duodenum (2), ileum (3), cecum (3) and sigmoid colon (3).

Fixation of the biopsy specimen of the mucous layer was carried out in a 10% solution of neutral formalin for 24 hours, followed by embedding in paraffin. Paraffin sections of 3–5 microns thick were prepared using a microtome. The sections were fixed onto glass slides coated with an adhesive (polylysine, APES) and incubated in a thermostat at 37° C for 12 hours. Then the sections were dewaxed and dehydrated in a battery of 3 xylenes, 2 absolute alcohols, 2 95% alcohols, 80% and 70% alcohol and distilled water.

In the obtained biopsies, the level and localization of expression of IL-2, IL-10, TNF-α, claudins-2, -3, and -5 were determined. Monoclonal antibodies to IL-2, IL-10, TNF-α and claudins 2,3,5 (IL-2 (1:100, Thermo Scientific #AHC0722), IL-10 (1:100, Thermo Scientific #AHC9102), TNF-α (1:100, Thermo Scientific #AHC3612), claudin 2 (1:100, Thermo Scientific #32–5600), claudin 3 (1:100, Thermo Scientific #PA5-32353, claudin 5 (1: 100, Thermo Scientific #35–2500) were used. The incubation time with primary antibodies was 60 minutes. At the end of incubation, the sections were washed in phosphate buffer (pH 7.0–7.6) and treated with secondary antibodies. Then the sections were incubated with secondary antibodies in humid chambers for 30 min, then washed in phosphate buffer (pH 7.0–7.6). An avidin-biotin complex (ABK KIT, DAKO #X0590) was used to label secondary antibodies. To visualize the binding site of the antibody with the antigen, a label was used—an enzyme, horseradish peroxidase, in the presence of a substrate—hydrogen peroxide—and a colorimetric reagent with 3,3-diaminobenzidine (LSAB, Dako Cytomation #K067811). As a result, the end product of the reaction, insoluble in organic solvents, was formed, which was visualized as brown staining of cell structures. Then the slides were rinsed in distilled water and the nuclei were tinted with hematoxylin. Then the glasses were moved over a battery of distilled water, 70% alcohol, 80% alcohol, 2 95% alcohols, 2 absolute alcohols, and 3 xylenes. After that, the sections were embedded in a synthetic environment using coverslips. To contrast the nuclei, the preparations were stained with hematoxylin.

The results were derived with both standard quantitative and semi-quantitative methods, depending on the percentage of stained cell nuclei. The intensity of staining was determined by a 6-point scale: 2 points—up to 20% of stained cells; 4 points—from 20% to 40% of stained cells; and 6 points—more than 40% of stained cells. The localization and intensity of staining (membrane and diffusive cytoplasmic staining) for CLDs were separately evaluated.

### DNA isolation and 16S library preparation

Fecal samples from 21 patients with IBS and 10 healthy volunteers were collected. Total DNA was isolated using AmpliPrimeDNA-sorb-AM kit (NextBio, Russia) for clinical specimens, according to the manufacturer’s protocol. The isolated DNA was stored at -20°C. For qualitive and quantitative assessment of the isolated DNA we used NanoDrop 1000 equipment (Thermo Fisher Scientific, USA). 16S library preparation was carried out according to the protocol of 16S Metagenomic Sequencing Library Preparation (Illumina, USA), which is recommended for Illumina MiSeq sample prep. The first round of amplification of V3-V4 16S rDNA variable regions was performed using the following primers: forward (TCGTCGGCAGCGTCAGATGTGTATAAGAGACAG-CCTACGGGNGGCWGCAG) and reverse (GTCTCGTGGGCTCGGAGATGTGTATAAGAGACAG-GACTACHVGGGTATCTAATCC) [23]. These primers are aimed at the amplification of bacterial (more than 90%) but not archaeal (less than 5%) rRNA genes. The approximate size of target amplicons was 440–460 bp. The amplification program (Applied Biosystems 2720 Thermal Cycler, USA) was the following: 1) 95° C— 3m; 2) 30 cycles: 95° C— 30s; 55° C— 30s; 72° C— 30s; 3) 72°C— 5m; 4) 4°C.

The derived amplicons were purified with AgencourtAMPure XP (Beckman Coulter, USA) beads according to the manufacturer’s protocol. The second amplification round was used for double-indexing samples with a combination of specific primers. The amplification program was the following: 1) 95° C— 3m; 2) 8 cycles: 95° C— 30s; 55° C— 30s; 72° C— 30s; 3) 72°C— 5m; 4) 4°C.

The purification of products of the second PCR round was also carried out using AgencourtAMPure XP. Concentration of the derived 16S rDNA libraries was measured using Qubit 2.0 fluorimeter (Invitrogen, USA) using Quant-iT dsDNA High-Sensitivity Assay Kit. The purified amplicons were mixed equimolarly according to the derived concentration values. Quality of the libraries was evaluated using Agilent 2100 Bioanalyzer (Agilent Technologies, USA) and Agilent DNA 1000 Kit. Sequencing was carried out on MiSeq machine (Illumina) and MiSeq Reagent Kit v2 (paired-end reads, 2x250 nt).

### Bioinformatics analysis of 16S sequencing data. Statistical analysis

The obtained sequences of forward and reverse reads were merged using MeFiT and CASPER, before quality-trimming and filtering with DADA2. For the most samples more than 99.5% reads were successfully merged. Further, the merged reads were analyzed by the DADA2 package (a part of the Bioconductor project) for R [24]. The analysis included the following steps: 1) the primer sequences were removed using cutadapt; 2) the reads were filtered by quality; 3) error distribution models were derived based on read quality profiles; 4) the sequencing errors were corrected; 5) RSV (ribosomal sequence variant; an analog of OTU) sequences were obtained; 6) chimeric RSVs were eliminated. Next, taxonomic annotation of the derived RSVs was carried out using DADA2 and the GreenGenes 13.8 16S reference sequence database [25].

Statistical analysis was carried out using Statistica for Windows 10.0 (StatSoft Inc.). Qualitative characteristics were described using absolute and relative (%) indicators, and quantitative characteristics using the median and 95% confidence interval (median [95% CI]). For the evaluation of statistical significance of differences between two and more independent groups, we used Mann-Whitney and Kruskal-Wallis non-parametric tests, respectively. For qualitative indicators, the Chi-squared test and, when necessary, exact Fisher test were used. For the correlation analysis, we used Kendall’s tau approach. Analysis of variance (ANOVA) was used when comparing more than two groups with quantitative indicators. In some cases, p<0.05 was considered as the significant level. In multiple comparisons, the Bonferroni correction was used (p-value threshold is defined as p = 0.05/n, where n is the number of comparisons of the same data).

## Results

Main characteristics of the observed groups are summarized in Table 1. There were no significant differences in gender distribution and average age.

**Table 1:** 

Sign	IBS (n = 31)	Control (n = 10)	p-value
Age, years	32 [26,0; 41,5]	38 [34,25: 42,75]	0,25
Sex, n (%)			0,71
Men	10 (32%)	4 (40%)	
Women	21 (68%)	6 (60%)	
Medical history (years)	16,0 [6,9; 23,3]	-	

### The expression of pro-inflammatory cytokines in the mucosal biopsy specimens of IBS patients was significantly higher than in the control group

**Fig 1:**
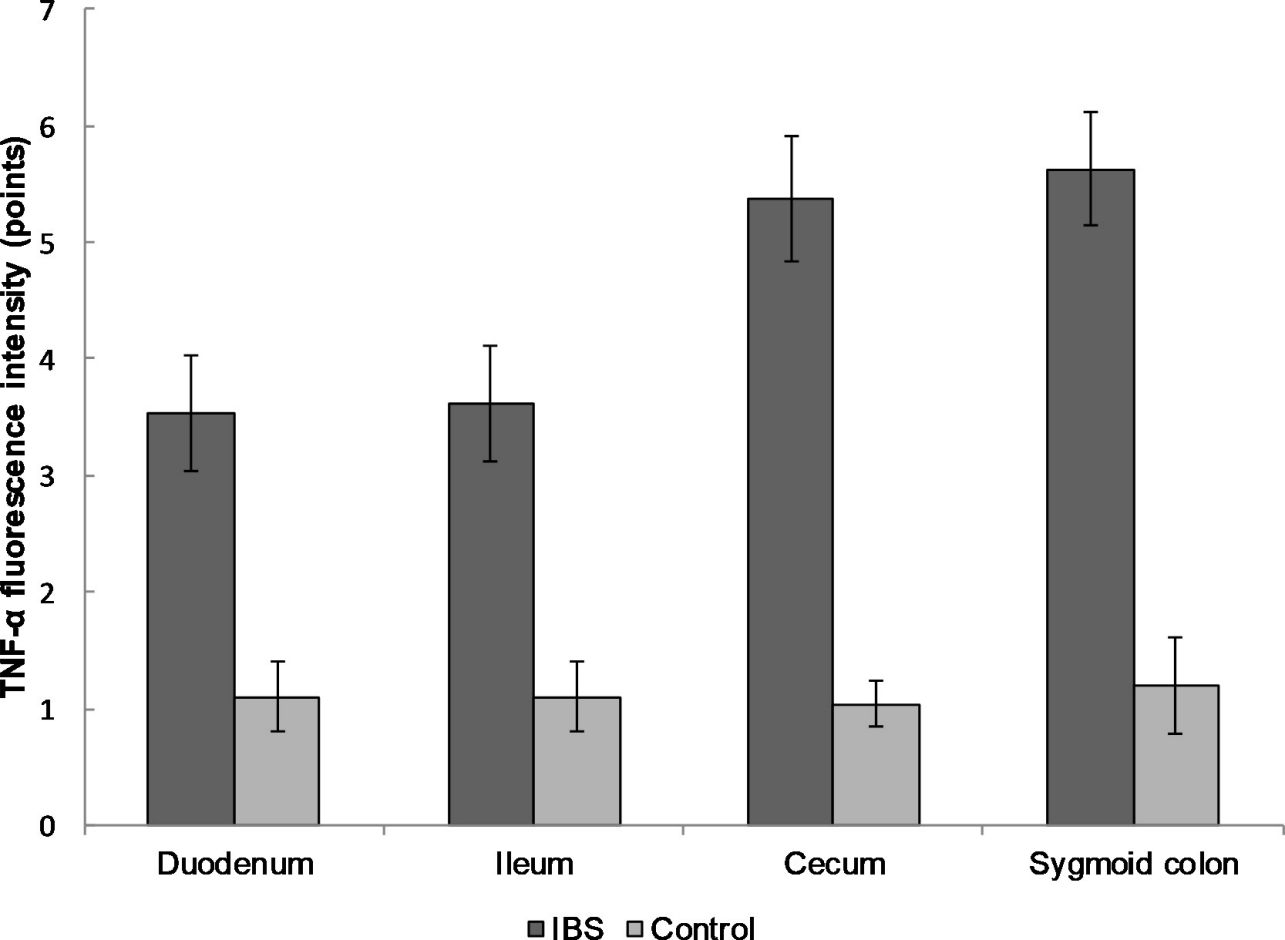
The expression of TNF-α in the mucosal biopsy of IBS patients and healthy control in different parts of the gastrointestinal tract. The expression of TNF-α gradually increased from duodenum to sigmoid colon compared to the control group. Mean values ± SD are shown. The statistical significance of the observed differences between IBS patients and healthy individuals was evaluated using Mann-Whitney U test (p-value < 0.0001 for all localizations).

The biopsies of the duodenum, ileum, cecum and sigmoid colon were performed in IBS patients and healthy volunteers. A statistically significant increase in the expression of the pro-inflammatory cytokine TNF-α was found in the mucosal biopsy of IBS patients from duodenum compared to healthy individuals (3.5 ± 0.5 versus 1.1 ± 0.3 points, respectively), ileum (3.6 ± 0.5 and 1.1 ± 0.3, respectively), cecum (5.4 ± 0.5 and 1.0 ± 0.2) and sigmoid colon (5.6 ± 0.5 and 1.2 ± 0.3; p < 0.0001 for all localizations; Fig 1).

**Fig 2:**
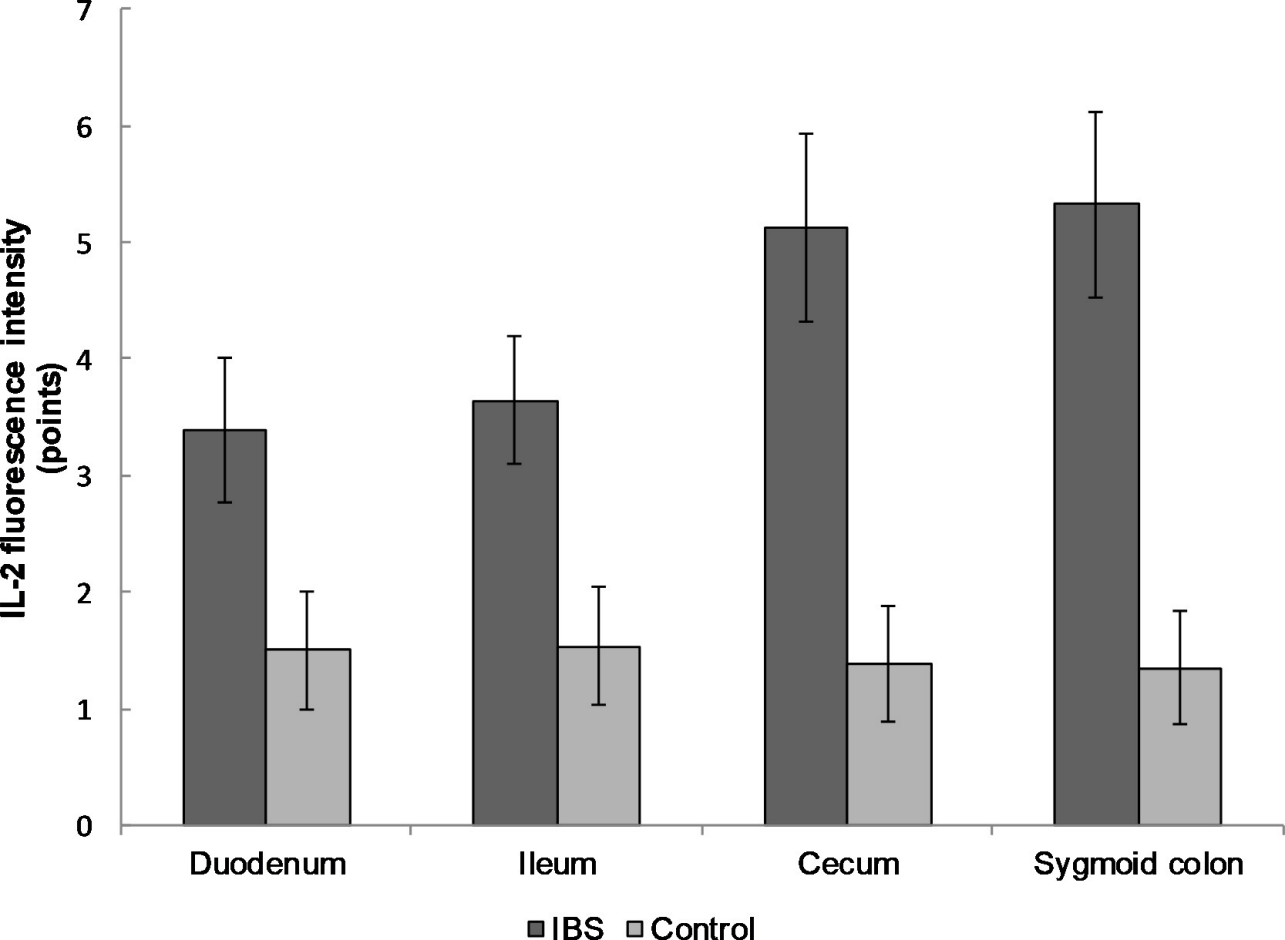
The expression of IL-2 in the mucosal biopsy of IBS patients and healthy control in different parts of the gastrointestinal tract. The expression of IL-2 gradually increased from duodenum to sigmoid colon compared to the control group. Mean values ± SD are shown. The statistical significance of the observed differences between IBS patients and healthy individuals was evaluated using Mann-Whitney U test (p-value < 0.0001 for all localizations). **The expression of anti-inflammatory cytokines in the mucosal biopsy specimens of IBS patients was significantly lower than in the control group**.

The expression of the pro-inflammatory cytokine IL-2 in the mucosal biopsy of IBS patients was higher than in healthy volunteers in duodenum (3.4 ± 0.6 versus 1.50 ± 0.51 points, respectively), ileum (3.7 ± 0.6 and 1.5 ± 0.5), cecum (5.1 ± 0.8 and 1.4 ± 0.5), sigmoid colon (5.3 ± 0.8 and 1.3 ± 0.5; p < 0.0001 for all localizations; Fig 2).

**Fig 3:**
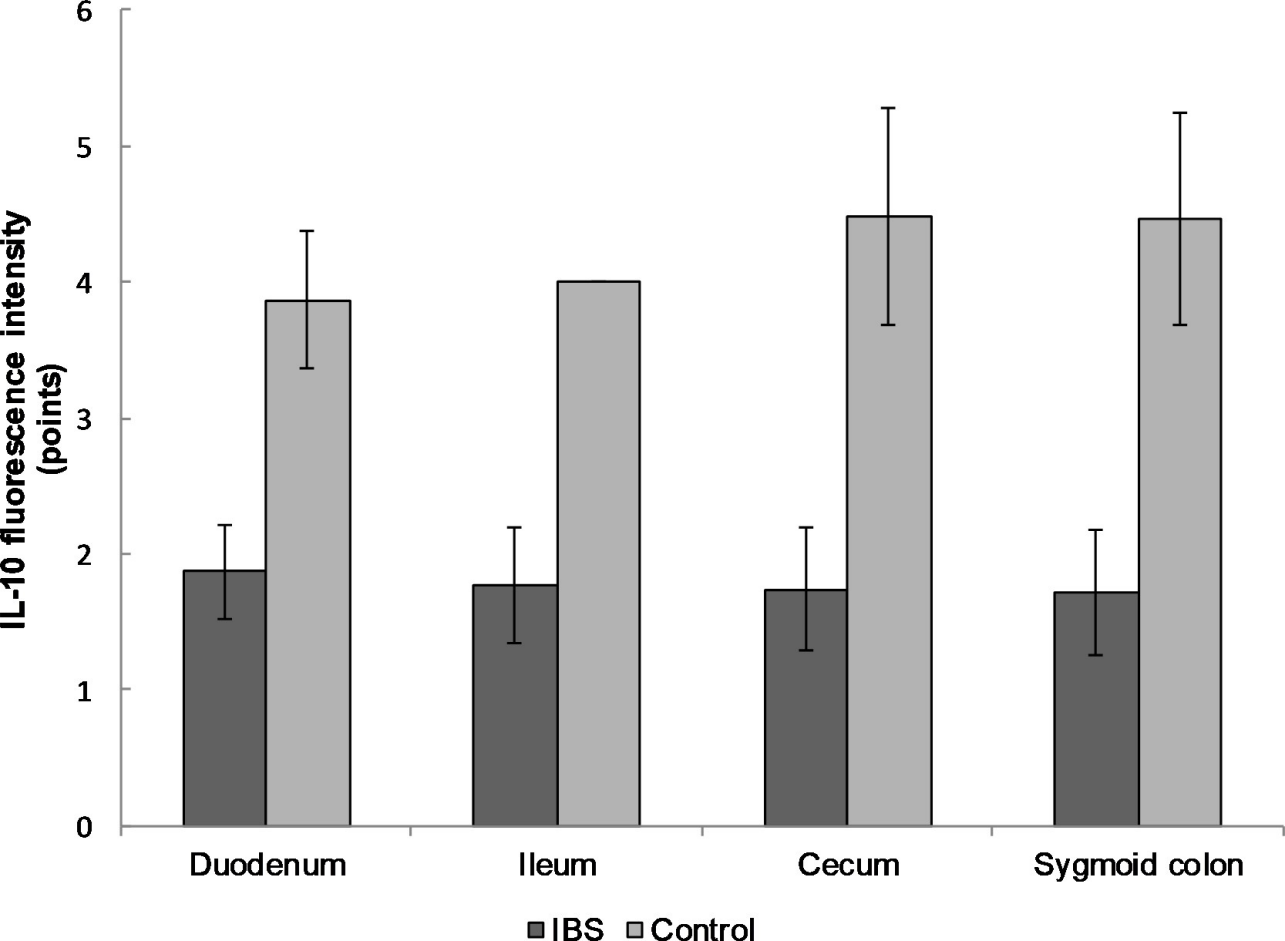
The expression of IL-10 in the mucosal biopsy of IBS patients and healthy control in different parts of the gastrointestinal tract. The expression of IL-10 was significantly lower in IBS group compared to the control group throughout the gastrointestinal tract. Mean values ± SD are shown. The statistical significance of the observed differences between IBS patients and healthy individuals was evaluated using Mann-Whitney U test (p-value < 0.0001 for all biopsy sites).

The expression of the anti-inflammatory cytokine IL-10 in the mucosal biopsy of IBS patients was significantly lower than in the control group also in all the examined biopsy sites: duodenum (1.9 ± 0.3 versus 3.9 ± 0.5 points, respectively); ileum (1.8 ± 0.4 and 4.0 ± 0.0); cecum (1.7 ± 0.4 and 4.5 ± 0.8), and sigmoid colon (1.7 ± 0.5 and 4.5 ± 0.8; p < 0.0001 for all localizations; Fig 3).

#### Changes in the tight junction proteins (CLD-2; 3; 5) expression in the mucosal biopsy specimens of IBS patients and control group

**Fig 4:**
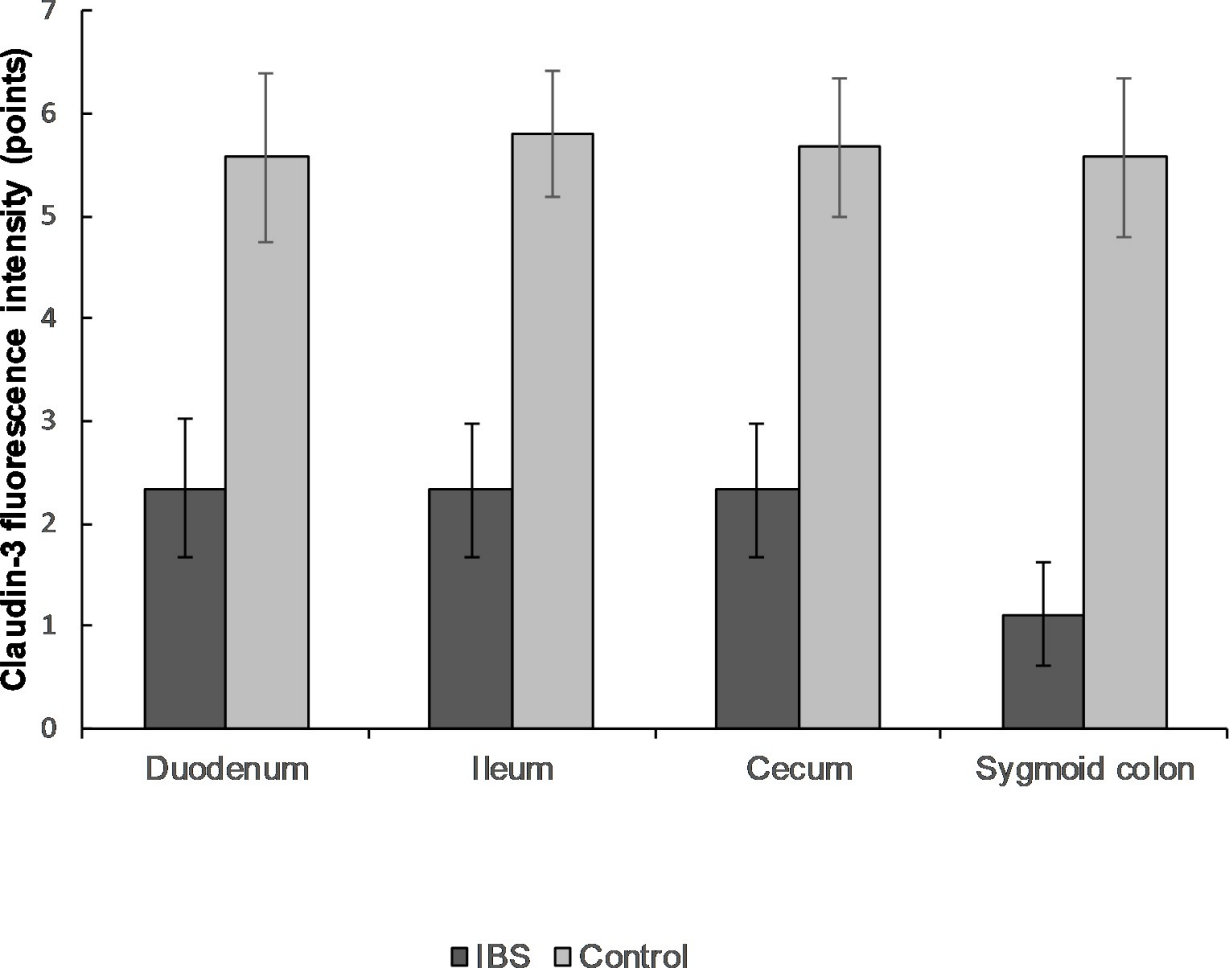
The expression of CLD-3 in mucosal biopsy of IBS patients and healthy control in different parts of the gastrointestinal tract. The expression of CLD-3 was significantly lower in IBS group compared to the control group throughout the gastrointestinal tract. Mean values ± SD are shown. The statistical significance of the observed differences between IBS patients and healthy individuals was evaluated using Mann-Whitney U test (p-value < 0.0001 in all cases).

**Fig 5:**
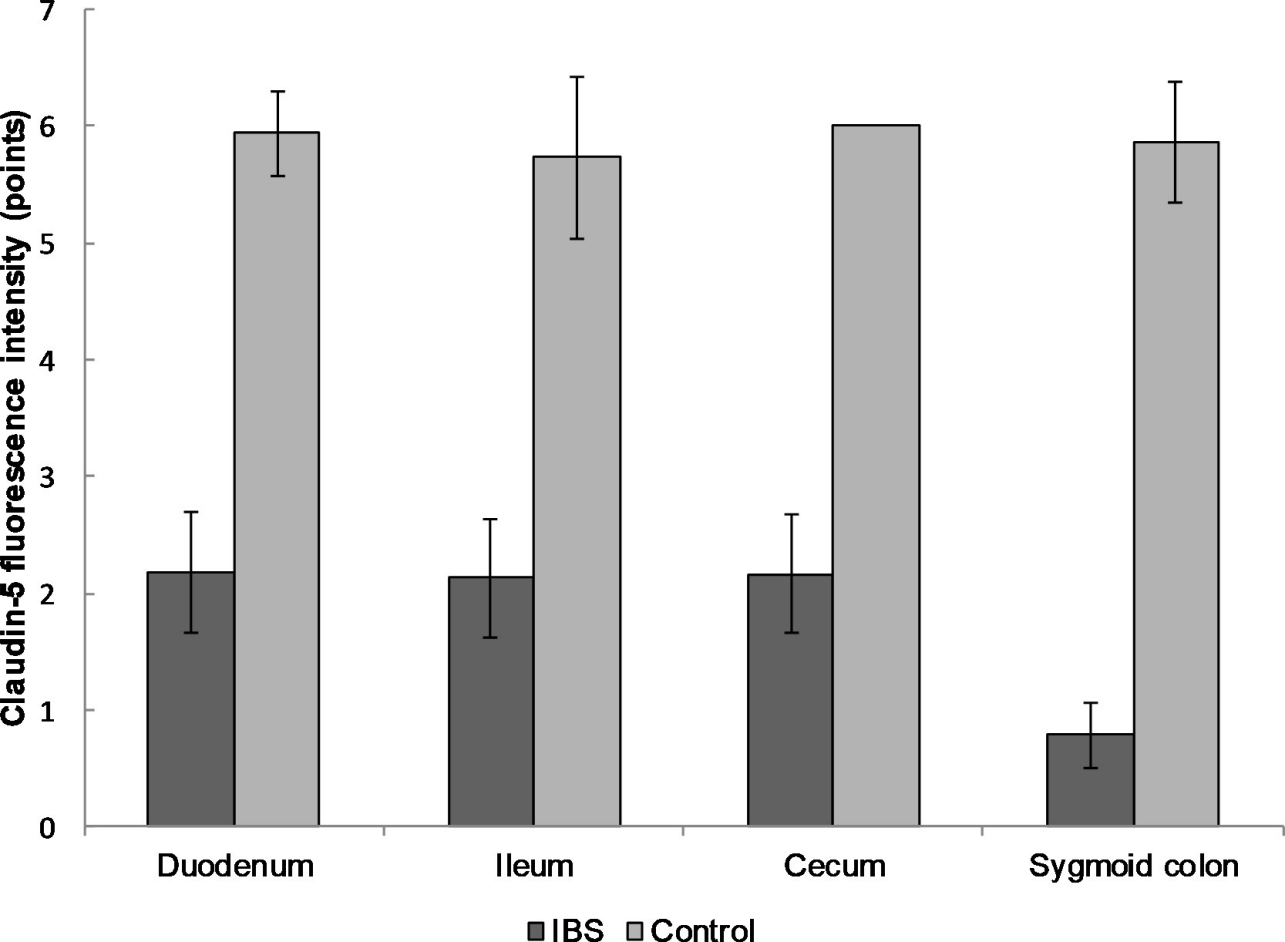
The expression of CLD-5 in the mucosal biopsy of IBS patients and healthy control in different parts of the gastrointestinal tract. The expression of CLD-5 was significantly lower in IBS group compared to the control group throughout the intestine. Mean value ± SD are shown. The statistical significance of the observed differences between IBS patients and healthy individuals was evaluated using Mann-Whitney U test (p-value < 0.0001 in all cases). There were no statistically significant changes in CLD-2 expression between groups.

Decreased expression of CLD-3 in the biopsy specimens of IBS patients was observed in all parts of the gastrointestinal tract: duodenum (2.3 ± 0.7 in IBS group and 5.6 ± 0.8 points in healthy persons), ileum (2.3 ± 0.6 and 5.8 ± 0.6, respectively), cecum (2.3 ± 0.6 and 5.7 ± 0.7), and sigmoid colon (1.1 ± 0.5 and 5.6 ± 0.8; p < 0.0001 for all localizations). The same results were observed for CLD-5 expression: duodenum (2.2 ± 0.5 versus 5.9 ± 0.4), ileum (2.1 ± 0.5 and 5.7 ± 0.7), cecum (2.2 ± 0.5 and 6.0 ± 0.0), sigmoid colon (0.8 ± 0.3 and 5.9 ± 0.5; p < 0.0001 for all localizations; Figs 4 and 5).

### The gradient of cytokines and tight junction proteins expression in the mucosal biopsy IBS patients and in control group

**Fig 6:**
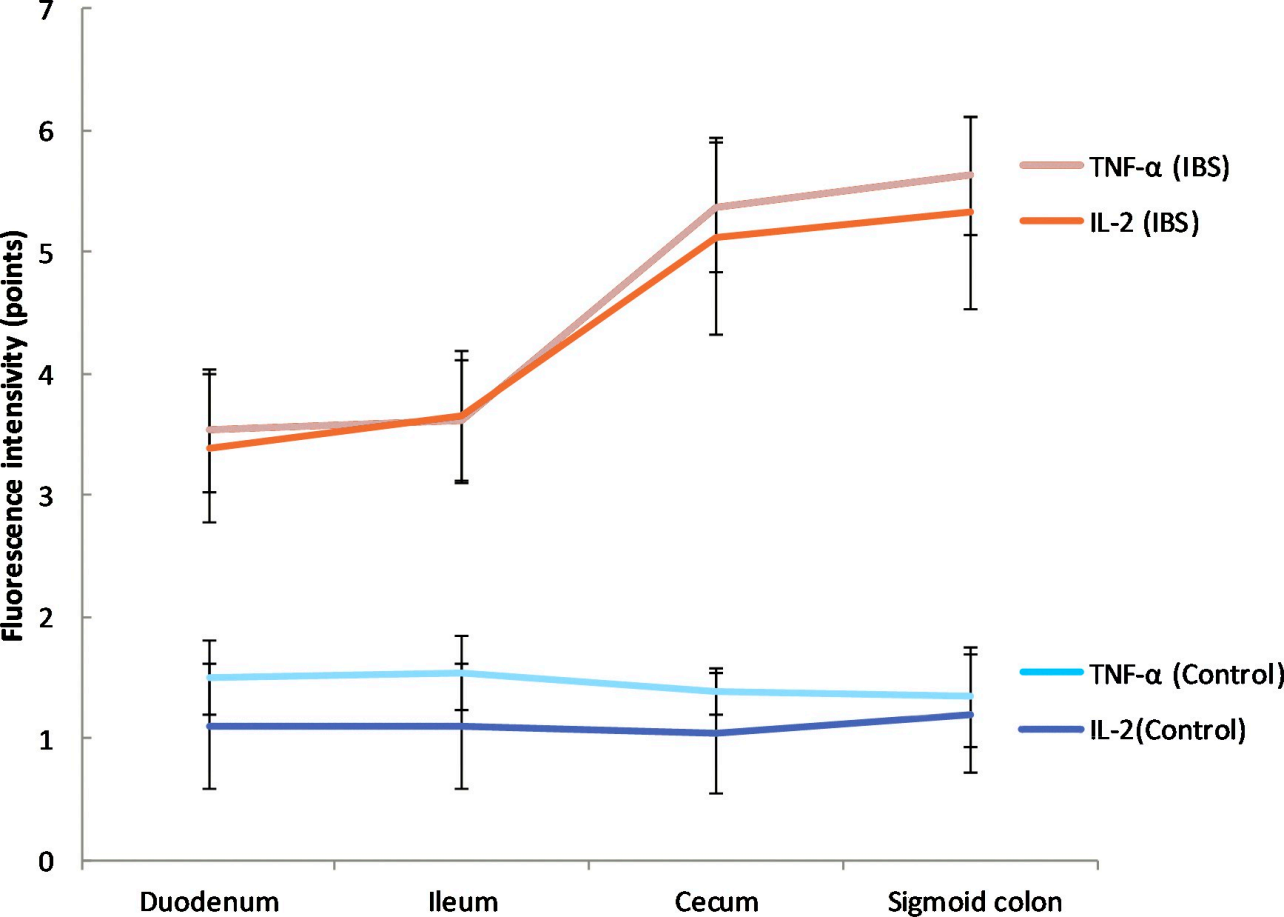
The gradient of cytokines and tight junction proteins expression in IBS patients and in control group. Positive gradient of expression of TNF- α and IL-2 in IBS group. *stands for TNF-α data, where p<0.05; # stands for IL-2 data, where p < 0.05.

The expression level of pro-inflammatory cytokines increased gradually from duodenum to sigmoid colon in IBS group, while in control it remained the same (Fig 6). Statistically significant changes in TNF- α expression were observed between ileum and duodenum and ileum and cecum (p<0.05), for IL-2 between ileum and duodenum (p<0.05).

### Qualitative and quantitative analysis of intestinal microbiota composition based on 16S rRNA gene sequencing revealed significant changes between IBS patients and healthy individuals

As a result of sequencing of 16S rRNA genes of fecal microbiome taken from 31 IBS and 10 healthy volunteers, a total of 12267 ribosomal sequence variants (RSV) were inferred. Among them, 4716 (62%) RSVs were treated as potentially chimeric (they correspond to 4.6% reads) and were excluded from the further analysis. The percentage of reads annotated at various taxonomic levels decreased from >99% (at the phylum level) to 74% [57%; 96%] (at the genus level)

**Fig 7:**
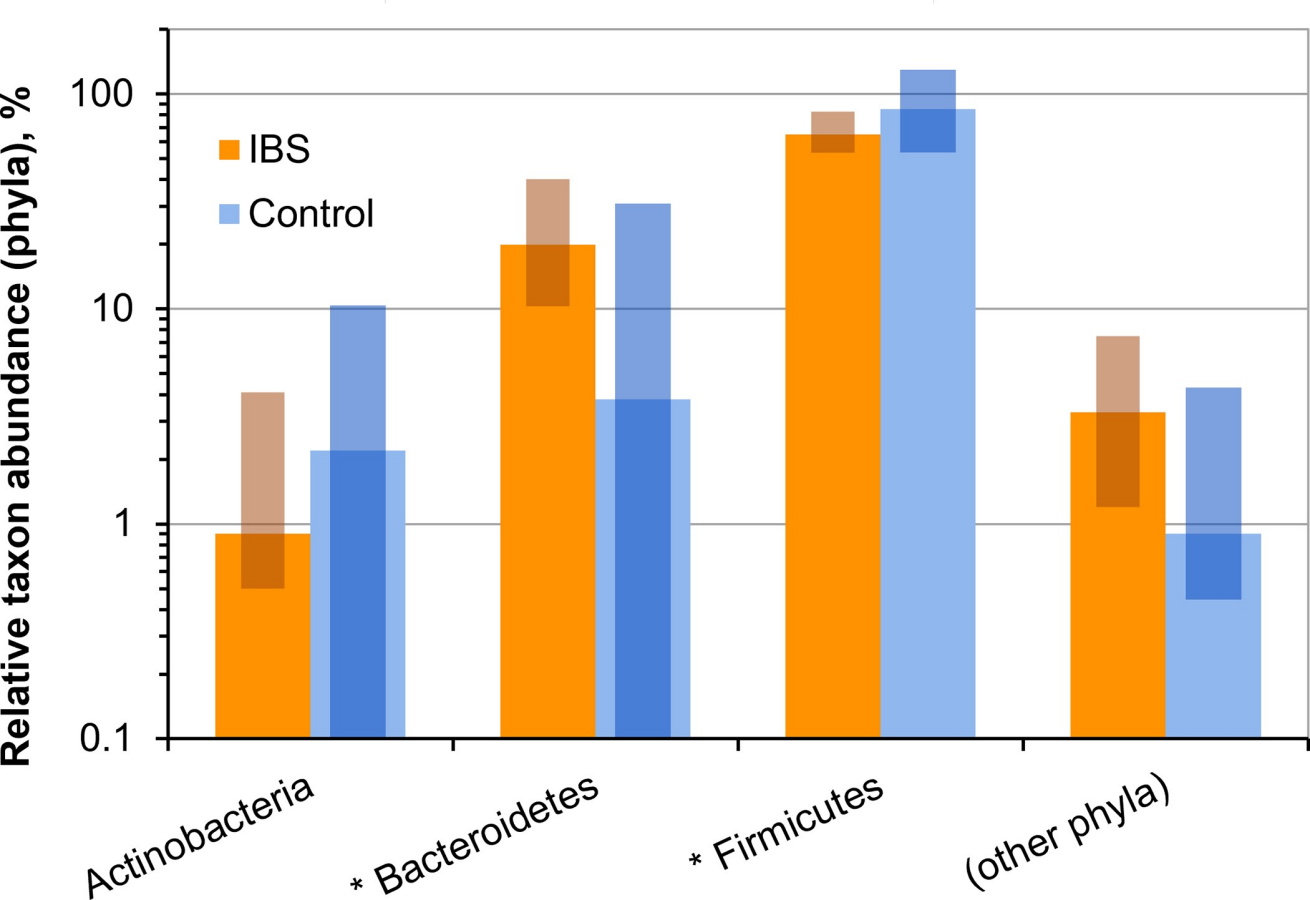
Relative abundance of bacterial phyla in the cohorts of IBS patients and healthy individuals. Solid bars correspond to median values; semi-transparent rectangles correspond to 95% confidence interval. The vertical axis is log-transformed. Asterisks (*) indicate cases for which p < 0.05.

At the phylum level, the analysis of taxonomic composition of intestinal revealed, the increased ratio of *Bacteroidetes* and decreased amount of *Firmicutes* in IBS patients, compared to healthy control (19.9 [10.3; 40.2] and 3.8 [0.0; 22.9], respectively), (65.0 [53, 3; 82.8] and 85.0 [72.3; 96.1]; p < 0.05 for both phyla). There were no significant differences in the relative amounts of *Actinobacteria* and *Proteobacteria* between these two groups (Table 2; Fig 7).

**Table 2:** Intestinal microbiota composition in IBS patients and healthy volunteers at the phylum level.

Phyla	IBS patients	Healthy control	p
*Actinobacteria*	0,9 [0,5; 4,1]	2,2 [0,0; 7,7]	0,160
*Bacteroides*	19,9 [10,3; 40,2]	3,8 [0,0; 22,9]	0,041*
*Firmicutes*	65,0 [53,3; 82,8]	85,0 [72,3; 96,1]	0,047*
*Proteobacteria*	3,3 [1,2; 7,5]	0,9 [0,6; 3,2]	0,210

Considering fecal microbial alpha diversity, ideally, the Chao1 index should reflect the species diversity of the microbiome (and Shannon index–both its diversity and balance), i.e. it should be calculated at the species level. Nevertheless, in most cases, we can really operate only at the RSV or genus/family level because of very poor annotation of reads at the species level. However, each RSV does not correspond to each species of bacteria. The number of RSVs may be either less than the real number of species (when sequencing too small fragments of the rRNA genes) or, as a rule, much greater than the actual number of species (because of presence of non-corrected sequencing errors, non-eliminated chimeras) [26]. Therefore, since we are mainly interested in comparing diversity between patient groups, we based our conclusions on Chao1 and Shannon indices at the genus level.

**Fig 8:**
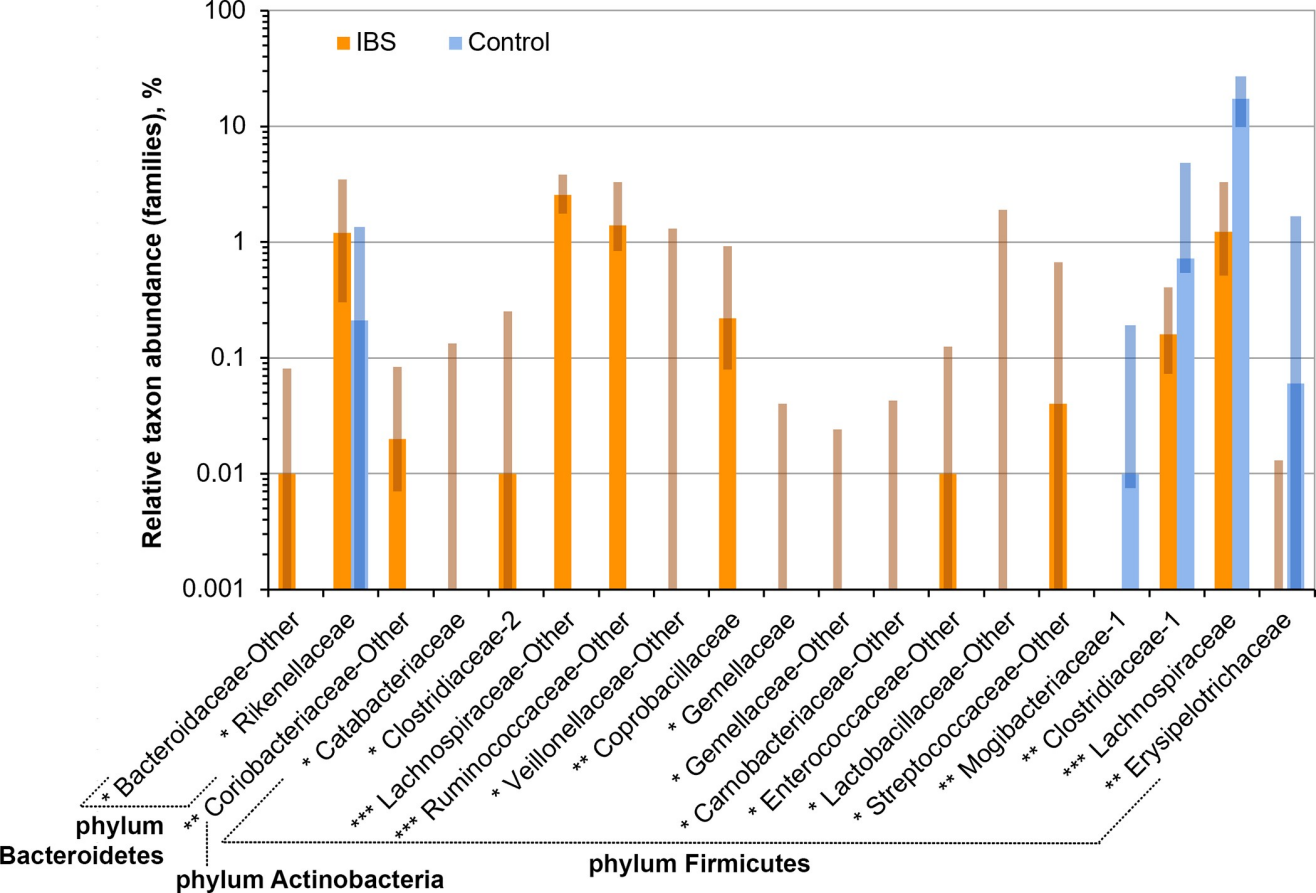
Relative abundance of bacterial families, for which statistically significant differences between the cohorts of IBS patients and healthy individuals (GreenGenes nomenclature). The degree of statistical significance is indicated with asterisks (*p < 0.05; **p < 0.005; ***p < 0.0005). Solid bars correspond to median values, semi-transparent rectangles correspond to 95% confidence interval. The vertical axis is log-transformed. Despite the fact that the plot shows a large number of *Firmicute* families that increase their abundance in IBS patients, there are a general decrease in the proportion of Firmicutes in IBS patients, mainly due to a significant drop in the abundance of the dominant *Lachnospiraceae* family.

**Fig 9:**
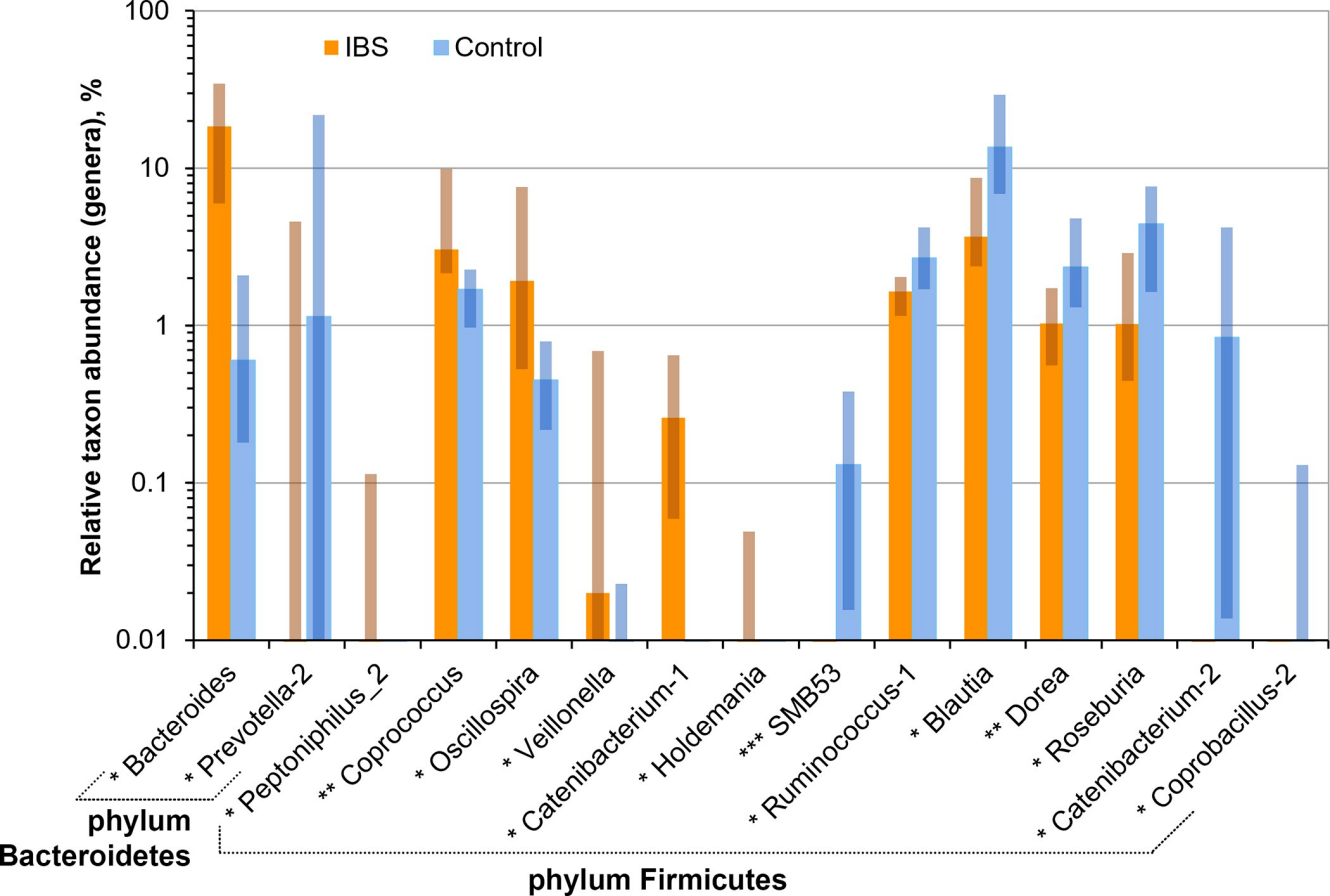
The relative abundance of bacterial genera in the cohorts of IBS patients and healthy individuals. The degree of statistical significance is indicated with asterisks (*p < 0.05; **p < 0.005; ***p < 0.0005). Solid bars correspond to median values, semi-transparent rectangles correspond to 95% confidence interval. The vertical axis is log-transformed.

The values of Shannon index for IBS patients (1.86 [1.17; 2.89]) tended to be lower than for healthy individuals (2.19 [1.38; 2.64]), but the difference was not statistically significant (*p* = 0.2) mainly because of the limited cohort size. We noticed the same tendency at the family and RSV levels. As it was expected, the values of the indices calculated at the family level were lower (and at RSV level–higher) than at the genus level (Figs 8 and 9). We noticed that the values of Chao1 indices were strongly correlated with the reads counts (Spearman’s rank correlation coefficient *r* = 0.67, *p* < 0.001), whereas Shannon index values demonstrated only slight correlations (*r* = 0.15, *p* = 0.02). The strong correlations between Choa1 index and reads counts may indicate: a) the excessive number of RSVs (because of sequencing errors/chimeras) or b) presence of lowly abundant taxons, which were not completely covered with the current sequencing depth. Since, in these cases it is better to consider only Shannon index values calculated at the genus level.

## Discussion

Despite the extensive research, etiology and pathophysiology of IBS remain incompletely understood. Proposed mechanisms involved in the pathogenesis include increased intestinal permeability [27], changes in the immune system reactivity [28], and gut microbiota composition [29, 30], visceral hypersensitivity, and impaired gut motility [31, 32].

**Fig 10:**
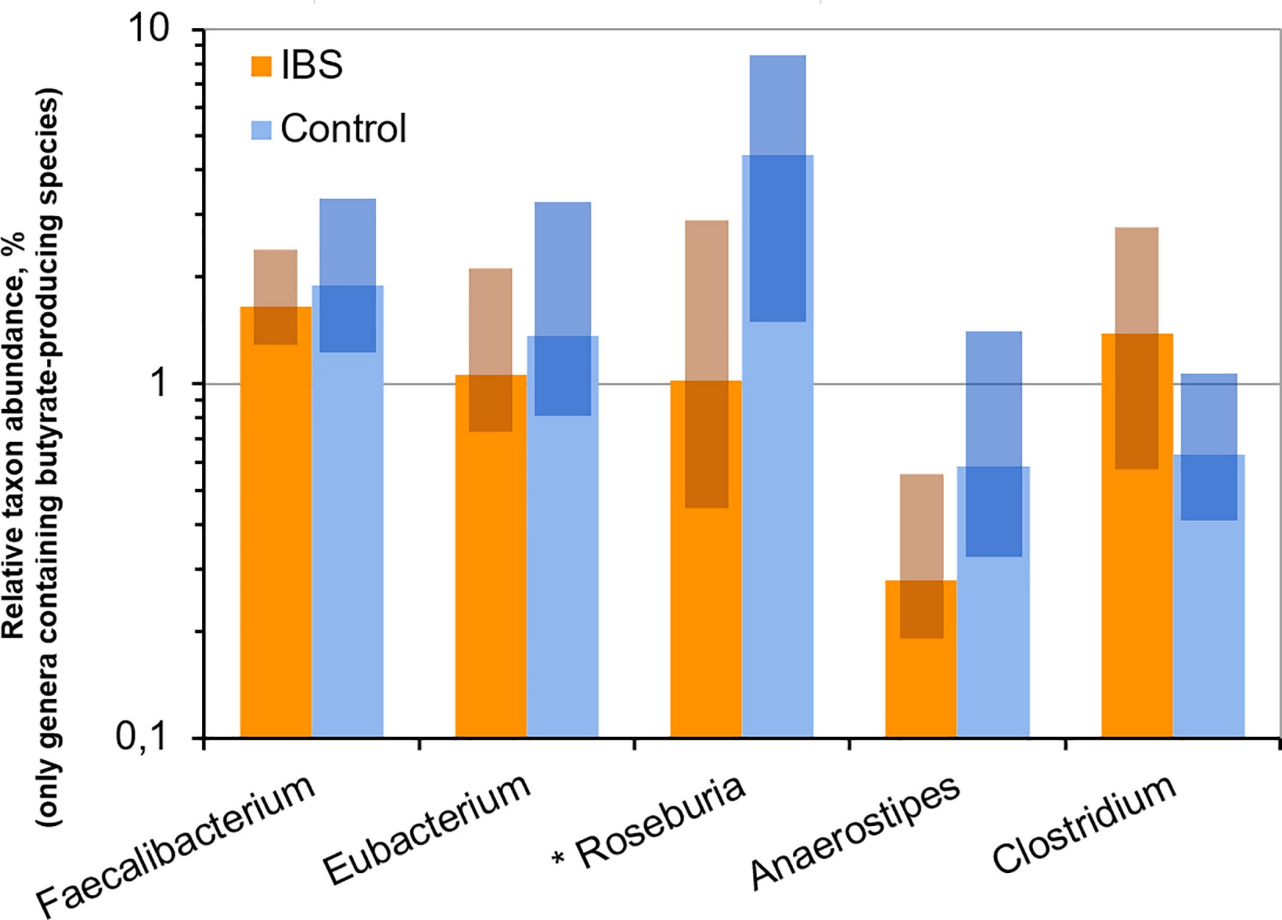
Relative abundance of bacterial genera, which may include butyrate producers, in the cohorts of IBS patients and healthy individuals. The degree of statistical significance is indicated with asterisks (*p < 0.05). Solid bars correspond to median values; semi-transparent rectangles correspond to 95% confidence interval. The vertical axis is log-transformed.

Disruption of the intestinal barrier permeability due to a decrease of membrane protein expression leads to the inflammatory response of the intestinal wall, which results in changes in gut sensitivity and motility [2, 5, 33, 34]. Inflammatory changes in the intestinal wall may also contribute to an increase in the number of *Bacteroidetes* in the intestinal microbiota composition [20]. Bacteroides, in turn, can dissolve mucosal glycoproteins [35], aggravate the intestinal barrier permeability due to the splitting of dense contact proteins [36]. On the other hand, previously it was shown that SCFAs modulate the expression of claudines 3, 4 and occludin, which are capable of increasing the barrier properties of the epithelium [37, 38]. The predominant bacteria that produce SCFAs in human microbiota are classified as family *Ruminococcaceae* (cluster IV) and genus *Eubacterium* (cluster XIVa), both are included in the order *Clostridiales*, class *Clostridia*, phylum *Firmicutes* [39]. Our study supports this hypothesis, according to the obtained data the abundance of butyrate producing bacteria is lower in IBS patients than in healthy individuals (Fig 10).

The low abundance of SCFA-producing bacteria in intestinal microbiota may exacerbate existing impaired permeability and promote immune system activation [38]. The mast cells degranulation causes release of inflammatory mediators (histamine, serotonin and proteases) resulting in lymphocyte activation and cytokine imbalance [40]. Patients with IBS were found to have higher levels of proinflammatory IL-6, IL-8, IL-1β, TNF-α and lower levels of anti-inflammatory IL-10 in both serum and intestinal mucosa [41, 42]. In addition, mast cells degranulation was shown to reduce the expression of tight junction proteins, probably through tryptase release [32].

Thus, the combination of disturbed intestinal permeability and altered composition of the intestinal microbiota lead to the developing of IBS symptoms. The expression gradient of pro-inflammatory cytokines (TNF- α and IL-2) may be associated with an increase in the number of microbial cells in the distal part of colon [43].

We admit that this study has some limitations such as the lack of direct gut permeability measurement, precise analysis of gut microbiota for patients with different variants and duration of IBS. A broader cytokine panel would be useful for a more detailed analysis of gut inflammation. Also, up to date, no data exists on how disease severity or duration can influence the intestinal microbiome, permeability, and degree of intestinal wall inflammation. Larger cohorts of patients to examine would be beneficial for future studies. The study of the tight junction proteins expression, the composition of the intestinal microbiota, the degree of inflammation in the intestinal wall, and their correlation with different duration of IBS history and symptoms severity may allow us to use them as biomarkers, and create a new approach for the therapy.

## Conclusion

Our study confirms the results of previous articles related to the role of impaired gut permeability and changes in the composition of the intestinal microbiota in patients with IBS. However, the relationship between these two pathogenetic mechanisms has not been studied before. A better understanding of the pathogenesis of IBS will improve the diagnosis and treatment of these patients, but further research including larger patient’s cohorts and a more detailed analysis of the gut microbiota is needed.
